# The Anatomy of the Human Body

**Published:** 1844-12

**Authors:** 


Art. VI.—*The Anatomy of the Human Body.* By J. Cruveilhier, Professor of Anatomy to the Faculty of Medicine of Paris; Physician to the Hospital of Salpetriere, and President of the Anatomical Society of Paris. The first American, from the last Paris Edition. Edited by Granville Sharpe Pattison, M.D., Professor of Anatomy in the University of New York; Member of the Medico-Chirurgical Society of London, etc., etc., etc. New York: Harper & Brothers. 1844. 8vo. p.p. 907.

Competent judges have pronounced this the ablest work on descriptive anatomy extant, and we feel no disposition, after the best examination we have been able to give it, to dissent from this opinion. Few men in the profession rank higher than Cruveilhier, and as an anatomist, healthy or morbid, his authority would every where be received as the best. The Messrs Harper have now made his system of anatomy accessible to the medical profession in this country, and have put it at a price less than one-fourth of the cost of the English edition. In this they have done the profession a service, and the cheapness of the work, added to its acknowledged superior excellence, it may be expected, will cause it to be introduced very generally into the libraries of our physicians.

The editor does not profess to have added much to the elaborate stores of Cruveilhier. In the first place, he says, the work is so perfect as not to require any additions from his pen, and, besides, is so voluminous, that any increase in its size would have diminished its value. But apart from this, "the editor has ever thought that an independent mind will shrink from mixing up and incorporating his thoughts with those of another."

Professor Pattison seems to consider an apology due from him to the profession, for engaging in the humble task of editing this work, instead of publishing a system of his own. This, he thinks, his position in the "*Metropolitan University of the United States,*" gave them a right to expect from him. The reason why he has not hitherto brought out such a work, is an apprehension that, since it would contain the peculiarities which belong to his lectures—namely, the application of anatomy to physiology and pathology—it would have the effect of rendering his lectures less interesting than they are at present to his pupils. Hence, he adds, he is determined nev er to "publish an original work on anatomy, so long as he is engaged in the duties of teaching."

The preface of the editor in proportion to the size of the volume is short, but for a preface it is long, and contains abundant proof of that spirit of jealousy, which appears to be increasing between the medical gentlemen of the two great Atlantic cities. We make no comments on the tone which characterizes this preface; but we must take the liberty of suggesting to the veteran professor, in future editions of the work, to have more care in the composition of the few pages in which he introduces it to his readers. Such glaring faults as mark many of the sentences in these four pages, might easily be avoided by a little attention. Nothing but the most extreme haste or negligence can explain the perpetration of such blunders as appear in the following sentence:

"New York is the great metropolis of the Union, and must very soon become like London and Paris, however distasteful it may be to those who have other interests, *become* the great centre, not only for medical publications, but also of medical education."

We say nothing about the good taste, or the bad taste, of introducing such a boast into a work on anatomy; but the construction of the sentence, it must be admitted, is any thing but tasteful. This, however, does not detract from the great merits of Cruveilhier's work. They speak for themselves, and will be appreciated by every one who is so fortunate as to obtain possession of the volume. Y.

